# Co-designed weight management intervention for women recovering from oestrogen-receptor positive breast cancer

**DOI:** 10.1186/s12885-022-10287-y

**Published:** 2022-11-22

**Authors:** J. M. Saxton, K. Pickering, S. Wane, H. Humphreys, H. Crank, A. S. Anderson, H. Cain, J. Cohen, R. J. Copeland, J. Gray, J. Hargreaves, R. J. Q. McNally, C. Wilson

**Affiliations:** 1 School of Sport, Exercise & Rehabilitation Sciences, Faculty of Health Sciences, University of Hull, Cottingham Road, Hull, HU6 7RX UK; 2 Advanced Wellbeing Research Centre, Sheffield Hallam University, Olympic Legacy Park 2 Old Hall Rd, Sheffield, S9 3TU UK; 3 Department of Sport, Exercise and Rehabilitation, Northumbria University, City Campus, Newcastle-Upon-Tyne, NE1 8ST UK; 4 Academy of Sport and Physical Activity, Sheffield Hallam University, Sheffield, S10 2BP UK; 5 Division of Population Health and Genomics, Centre for Research Into Cancer Prevention and Screening, Ninewells Hospital and Medical School, University of Dundee, Dundee, DD1 9SY UK; 6 Newcastle Upon Tyne Hospitals NHS Foundation Trust, Queen Victoria Road, Newcastle Upon Tyne, NE1 4LP UK; 7 York Medical School, Hull Heath Trials Unit, University of Hull, Cottingham Road, HullHull, HU6 7RX UK; 8 Department of Nursing, Midwifery Are Health, Northumbria University, Coach Lane Campus, Newcastle Upon Tyne, NE7 7XA UK; 9 Carnegie School of Sport, Leeds Beckett University, 9 Fairfax Hall, Headingley Campus, Leeds, LS6 3QS UK; 10 Population Health Sciences Institute, Newcastle University GB, Newcastle Upon Tyne, NE1 7RU UK; 11 Department of Oncology and Metabolism, The Medical School, University of Sheffield, Beech Hill Road, Sheffield, S10 2RX UK

**Keywords:** Breast cancer, Overweight, Obesity, Co-design, Weight loss intervention

## Abstract

**Background:**

Weight gain is commonly observed during and after breast cancer treatment and is associated with poorer survival outcomes, particularly in women with oestrogen receptor-positive (ER +) disease. The aim of this study was to co-design (with patients) a programme of tailored, personalised support (intervention), including high-quality support materials, to help female breast cancer patients (BCPs) with ER + disease to develop the skills and confidence needed for sustainable weight loss.

**Methods:**

ER + BCPs were recruited from two UK National Health Service (NHS) Trusts. The selection criteria included (i) recent experience of breast cancer treatment (within 36 months of completing primary treatment); (ii) participation in a recent focus group study investigating weight management perceptions and experiences; (iii) willingness to share experiences and contribute to discussions on the support structures needed for sustainable dietary and physical activity behaviour change. Co-design workshops included presentations and interactive activities and were facilitated by an experienced co-design researcher (HH), assisted by other members of the research team (KP, SW and JS).

**Results:**

Two groups of BCPs from the North of England (*N* = 4) and South Yorkshire (*N* = 5) participated in a two-stage co-design process. The stage 1 and stage 2 co-design workshops were held two weeks apart and took place between Jan–March 2019, with each workshop being approximately 2 h in duration. Guided by the Behaviour Change Wheel, a theoretically-informed weight management intervention was developed on the basis of co-designed strategies to overcome physical and emotional barriers to dietary and physical activity behaviour change. BCPs were instrumental in designing all key features of the intervention, in terms of *Capability* (e.g., evidence-based information, peer-support and shared experiences), *Opportunity* (e.g., flexible approach to weight management based on core principles) and *Motivation* (e.g., appropriate use of goal-setting and high-quality resources, including motivational factsheets) for behaviour change.

**Conclusion:**

This co-design approach enabled the development of a theoretically-informed intervention with a content, structure and delivery model that has the potential to address the weight management challenges faced by BCPs diagnosed with ER + disease. Future research is required to evaluate the effectiveness of the intervention for eliciting clinically-important and sustainable weight loss in this population.

**Supplementary Information:**

The online version contains supplementary material available at 10.1186/s12885-022-10287-y.

## Background

Weight gain is commonly observed during and after breast cancer treatment and is associated with poorer survival outcomes, notably in women with oestrogen receptor-positive (ER +) disease [1, 2], which accounts for 70% of all incident cases [3]. Higher body fat levels increase the risk of disease recurrence amongst ER + breast cancer patients (BCPs) because of increased aromatase activity and circulating levels of oestrogens and androgens [1, 4]. Other risk factors for disease recurrence are also associated with excess body fat, including abnormal insulin and adipokine metabolism, impaired anti-tumour immunity and chronic low-grade systemic inflammation [1, 4].

This evidence provides a strong rationale for the development of interventions that can provide the weight management support women need after ER + breast cancer treatment. However, understanding and addressing the challenges women experience in engaging with weight management behaviours during and after ER + breast cancer treatment is an important step in designing effective interventions for sustainable weight loss. Important barriers to health behaviour change amongst women undergoing breast cancer treatment include treatment-related physical symptoms which impede physical functioning (e.g., lymphoedema which restricts upper-limb range of motion), fatigue, pain, lack of confidence, body image concerns, fears about health behaviour change due to feelings of vulnerability, co-morbidities and conflicting priorities (e.g., work commitments, family caring duties, etc.) and low motivation [5–8]. In addition, women commonly experience deficits in the availability of clear, simple and credible information on lifestyle-related issues, citing insufficient support from health professionals [8, 9].

Studies show that group-based interventions provide an opportunity for peer-to-peer support and a forum for addressing the anxieties and challenges women face after breast cancer, thereby helping to build the skills and confidence needed to increase engagement in healthy lifestyles. Successful group-based weight-loss interventions (providing support for dietary and physical activity behaviour change) have used a variety of delivery formats, including face-to-face workshops for 8–15 women alongside remote support methods such as telephone, emails, text-messaging and printed mail-outs [10–14]. In addition, the use of self-regulatory behavior change techniques (e.g., goal setting, self-monitoring), inclusion of an educational component, setting of graded tasks, and establishing a structure for frequent contact and social support is consistent with best-evidence strategies for promoting changes in dietary and physical activity behaviours in the general population [15] and in people living with and beyond cancer [16].

In the UK, support for health behaviour change after primary treatment for breast cancer is provided by prominent cancer charities but this is limited in scope and content [17, 18]. Offering a route to longer-term, tailored (bespoke), weight management support would therefore address an important unmet need for women and their treating clinicians at what is frequently an opportune ‘teachable moment’ for patients [19]. Drawing on published empirical evidence and guided by the UK Medical Research Council (MRC) Framework for Developing and Evaluating Complex Interventions [20] and the Person-Based Approach to Intervention Development [21], the aim of this study was to co-design (with patients) an accessible and adoptable weight loss intervention that prioritises the issues and concerns faced by women recovering from ER + breast cancer treatment. Co-design is a joint venture involving service users, healthcare professionals and researchers working together [22] with the aim of maximising the potential of an intervention, in terms of its impacts on health, policy and practice [23]. It provides valuable insights into the perspectives and psychosocial context of patients and has been used to develop accessible and adoptable interventions in other cancer populations [24, 25]. By applying this co-design method, we aimed to develop a programme of tailored, personalised support (including high-quality support materials) to help overweight BCPs build the skills and confidence needed to lose weight and maintain weight loss. Furthermore, to develop an intervention model that is scalable, dovetails with existing UK breast cancer care pathways, and brings about the step-change improvement in support for sustainable dietary and physical activity behavior change needed by many women after primary treatment for ER + breast cancer.

Use of a theoretical framework in the development of health behaviour change interventions is associated with improved effectiveness [26]. Intervention development was guided by the Behaviour Change Wheel (BCW), which comprises the COM-B model [27] and is supported by the Theoretical Domains Framework (TDF), the intervention functions matrices and a taxonomy of behaviour change techniques [28]. The COM-B model defines health behaviour change (B) in terms of changing one or more components of physical and psychological capability (C), the availability of physical and social opportunities (O) and automatic and reflective motivation (M). The TDF offers practical guidance for implementing interventions and was formed by grouping together constructs from a number of behaviour change theories (domains) which can be mapped onto the principal constructs of COM-B. This theoretical approach has been used previously as a framework for developing health behaviour change interventions in other cancer populations [24, 29, 30]. Here, we report on the process and outcomes emanating from the co-design workshops, with the latter being used to define the overall structure, content and delivery method of the intervention and associated resources.

## Methods

### Co-design method

A recent linked focus group study with BCPs and healthcare professionals (HCPs) explored the challenges of dietary and physical activity behaviour change during and beyond the breast cancer care pathway [31]. Four overarching themes (and 10 subthemes) were identified from the focus group study which weight management interventions for ER + BCPs should aim to address: (1) Treatment; (2) Support for lifestyle behaviour change; (3) Information availability for BCPs; (4) Knowledge of current evidence amongst HCPs. This linked focus group study constituted a behavioural diagnosis as a first step to identifying the COM-B and TDF domains that need to be targeted [27]. In contrast, co-design workshops aimed to explore strategies for overcoming barriers to weight management behaviours with BCPs and to get their ideas on how best to develop an intervention to support behaviour change, in terms of content, structure and delivery model. All participants provided written, informed consent prior to data collection and the study was approved by the Northwest Preston National Health Service (NHS) Research Ethics Committee (18/NW/0400).

### Participant recruitment

Female BCPs who were recruited to the previous focus group study in the initial phase of this research [31] were invited to engage in the co-design workshops. Two groups of BCPs each attended two co-design workshops in the North of England (*N* = 4) and South Yorkshire (*N* = 5). Women were eligible to participate in the study if they were over 18 years of age and were more than eight weeks since completion of chemotherapy (providing time to reflect on their experience of adjuvant treatment) and less than 36 months since completion of primary treatment for ER + breast cancer (for accurate recall of experiences), with a BMI ≥ 25 kg/m^2^. Women being prescribed hormone therapies were eligible.

### Procedures and data collection

Co-design workshops were implemented and structured as detailed below, with each workshop being approximately 2 h in duration, and including refreshments. They included presentations with interactive discussions, brainstorming sessions (including picture image prompts/* “How might we…?”* flipchart and sticky-note questions) aimed at identifying key weight management issues and developing focused solutions, and prototyping stations which provided examples of intervention tools and resources that might be of interest (Table 1). Activities were facilitated by an experienced co-design researcher (HH), assisted by two other members of the research team (KP and SW), who recorded and collated the data. All participants in each of the two groups of BCPs attended two co-design workshops, which were held two weeks apart, and took place between Jan–March 2019.

**Table 1:** Co-design workshop activities

**Co-design Workshop 1**	
**ACTIVITY/TASK**	**PURPOSE/AIM**
**Introductions and icebreaker**	Serve as a “Why are we here?” and establish mutual expectations
Write/draw a word(s) or picture(s) to summarise: *“If you were taking part in our future weight loss intervention, how you would like to feel at the end of the intervention?”*	
**Feedback from focus groups and ranking exercise**	Present back to participants the main concerns and challenges identified in the focus groups—in the context of what type of support is needed to address them
*Which physical and psychological side effects have the greatest impact on you being physically active or sticking to a healthy diet/changing your diet?* Rank the five most important challenges identified in stage 1	
**Factors influencing health behaviour change**	Continue to progress thinking and conversations regarding what components the intervention needs to include. Identify potential behaviour change strategies that will be helpful and effective from participants’ perspective. NB: The value of these ranking exercises is in the discussion they generate. Facilitators use opportunities to ask “why” people are ranking things higher or lower and explore differences of opinion. All this contributes to an increasingly nuanced understanding about what might work, why and for whom, and where variation/flexibility is needed to suit different people
Which behaviour change techniques would help you to engage with a supportive intervention/keep you involved? Discussion and ranking exercises in the context of side-effects and challenges identified in the focus groups, plus discussion of behaviour change techniques identified by research team	
**Picture/image association**	Provides a steer on the images/words that resonate with this population – informs design briefs for intervention materials; ensures conversations are light-hearted so as to not dwell on negatives of the breast cancer experience
Display keywords and images associated with the study aims (e.g. breast cancer, weight loss, success, achievement, support) and ask participants to discuss how these words and images make them feel	
***“How Might We…?”* flipchart questions**	*“How might we…?”* statements on flipcharts around the room are designed to reframe problems as opportunities and to encourage focused solutions without limiting creativity. Sticky note ideas/suggestions as the basis for group brainstorming/discussion to generate solutions/opportunities Statements based on issues identified in stage 1, earlier ranking exercises and evidence for effective behaviour change strategies from the literature. Flipcharts retained for analysis prior to co-design workshop 2
*HMW make it easier for you to attend the programme on days/weeks when you feel unwell/low?* *HMW convince you that the programme is worth taking part in?* *HMW help you to feel less self-conscious during physical activity sessions?* *HMW provide you with diet information and advice that is clear to understand but also allows you to have some choice and flexibility?* *HMW make it as easy as possible for you to attend or complete all the sessions of the programme?* *HMW ensure the programme provides social support?* *HMW make the programme fun?* *HMW help you to keep on track with your diet in between group sessions?* *HMW link parts of the programme to the side-effects of breast cancer you have told us are most troublesome?*	
**Co-design Workshop 2**	
**ACTIVITY/TASK**	**PURPOSE/AIM**
**Group discussion—unanswered questions from workshop 1**	Generate discussion to gather more information on how/why to include specific intervention components. Explore the implications of making certain features of the intervention ‘opt-in’ or ‘opt-out’
e.g. *How important is it that intervention support groups are homogenous (i.e. women of the same age, with similar experiences, issues and concerns, *etc*.)? Should there be optional elements to a weight loss intervention (i.e. setting-up social media support groups, socialising after support sessions, weekly group/individual goals, *etc*.)? How (and how frequently) should progress be tracked?* Practical questions (e.g. venue for support sessions, timing, etc.)	
**Prototyping stations**	Discussion and further refinement of resource prototypes to finalise what these should look like; consider how resources should be used in the intervention and discount any that become apparent as less useful or less desirable
As a group, move around three workstations set-up with templates, examples and very rough prototypes of intervention resources/printed support materials based on ideas and proposed solutions generated in co-design workshop 1. These were created/provided by the facilitators and/or brought in by participants as useful examples of what could work best (e.g. diaries containing recipes, goal setting templates, etc.). Discussions were clustered around three core themes: information sources and intervention materials; progress tracking and monitoring; maintaining motivation and support	
**Ask the Physiologist**	From Workshop 1, it was clear that participants want the intervention to be underpinned by best available evidence. They wanted better knowledge and understanding of the physiological mechanisms underpinning their cancer treatment, bodily changes and symptoms, and the importance of a healthy diet and physical activity in this context. Provides valuable insight into the topics needing to be covered in support sessions and intervention materials
The group participates in a question & answer session with an exercise physiologist who has experience of leading exercise and dietary intervention trials with cancer patients	
**Introduction to proposed intervention structure**	Group discussion to refine and update the intervention delivery model and consider what will work best and why. Gather views/ideas on delivery/receipt of the intervention to maximise interest and participation
A diagram presented by the research team to outline a proposed intervention delivery model, based on discussions in co-design workshops 1 and 2 and best evidence from the literature (e.g. frequency of support sessions, duration of the intervention, touchpoints for enhanced support, etc.)	

#### Co-design workshop 1

An initial co-design workshop presented key findings from the focus group study [31] and actively engaged BCPs in discussions with other BCPs and the workshop facilitators (co-design expert [HH], physiotherapist [SW], specialist cancer exercise practitioner [KP] and senior cancer survivorship researcher [JS]), which focused on addressing perceived barriers and challenges to weight management behaviours in the context of COM-B and TDF domains (Table 1). *How might we…”* statements were used to reframe problems and challenges as opportunities, encourage the development of creative solutions and provide important insights into adoptable intervention features.

#### Co-design workshop 2

A follow-on co-design workshop aimed to consolidate learning from the first workshop and further inform intervention design (Table 1). In the follow-on workshop, emphasis was placed on the types of information sources that participants would trust and find useful, the motivational support structures required and exploring perceptions on progress tracking and monitoring.

### Analysis

**Fig. 1:**
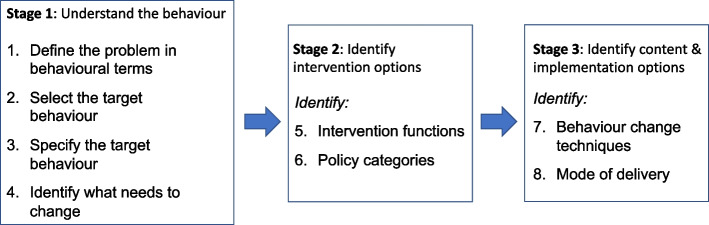
The behaviour change intervention design process. Taken from Michie S, Atkins L, West R. (2014) The Behaviour Change Wheel: A Guide to Designing Interventions [27]

The behaviour change intervention design process [27] was used as a framework to aid the development of the intervention, and consists of 8 steps (Fig. 1). The linked focus group study [31] aimed to understand the challenges BCPs face in adopting weight management behaviours and identifying the COM-B domains that need to be targeted. Emergent themes and subthemes were mapped onto a matrix combining the six COM-B sub-components (Physical and Psychological Capability, Physical and Social Opportunity and Reflective and Automatic Motivation). The focus of the co-design process was to identify what needs to change as a means of informing the development of an intervention guided by the TDF, e.g., what elements of a support programme would be essential to a weight loss programme and what elements would need to have an optional/flexible component. Intervention functions related to the identified COM-B and TDF domains were considered using the intervention function matrix [27] and the behaviour change taxonomy was used in specifying intervention content, enabling barriers and facilitators to be targeted [28]. Policy categories were considered in the context of NHS resource limitations and the timing of intervention implementation. Evidence from the linked focus group study [31] supported the dovetailing of this support programme with the end of primary treatment.

## Results

**Fig. 2:**
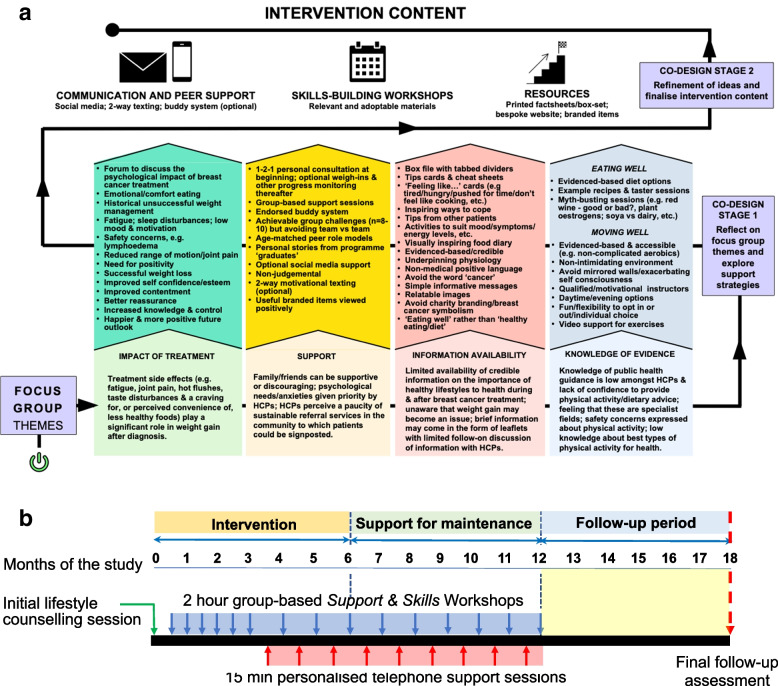
**a** Overall schema showing progression from the focus group stage through the co-design process. **b** Intervention delivery model

Participant characteristics are presented in Table 2. Key findings emanating from the co-design workshops, in the context of the COM-B domains and sub-domains are presented below. The mapping of intervention functions, behaviour change techniques and delivery modalities with the COM-B sub-components and TDF domains is shown in Table 3 and the overall structure, content and delivery method of the intervention and associated resources are presented in Fig. 2a and b and the Supplementary Table.

**Table 2:** Participant characteristics

BCPs recuited to the co-design workshop		
**N**	**9**	
	**Mean ± SD**	**Range**
Age (y)	51.6 ± 11.5	35–70
Months since treatment	9.8 ± 11.0	2–36
**Menopausal status at diagnosis**		
Pre	5	
Peri	1	
Post	3	
**Marital Status**		
Married	5	
Single	2	
Widowed	1	
Divorced	1	
**Education**		
Secondary school	3	
Vocational quilification	1	
University	5	
**Children**		
Yes	7	
No	2	
**Ethnicity**		
White	9	
**Treatment**		
Chemotherapy	7	
Radiotherapy	9	
Endocrine therapy	5	
Surgery	9

**Table 3:** COM-B and TDF domain mapping

COM-B	TDF domain	Intervention Function	Behaviour change technique	Mode of delivery and intervention content
Physical Capability	Physical Skills	Education, Training, Enablement	Graded task	Evidence-based physical activity/exercise efficacy and safety knowledge, e.g., lymphoedema and fatigue management; appropriate goal progression; supportive team challenges
Psychological Capability	Behavioural regulation	Education, Training, Enablement	Self-monitoring	Pedometers/apps; food and activity logs; reflective journals
	Knowledge	Education	Health consequences	Cooking skills guide; benefits of physical activity after cancer; portion size; snack advice; reading food labels; hydration; healthy eating/physical activity for management of side-effects and comorbidities
Physical Opportunity	Environmental context and Resources	Training, Enablement, Environment restructuring	Sign-posting; prompts	Physical activity/exercise taster sessions; sign-posting to local opportunities; on-line exercise videos; access to high-quality example meal recipes; cooking skills guides; food taster sessions Reminder text messages
Social Opportunity	Social influences	Modelling, Training, Enablement	Social support; social comparison; information about other’s approval	One to one contact with qualified lifestyle advisor; Online support; group-based support sessions; education for friends and family; social media support groups
Automatic Motivation	Emotion	Training, coercion, environmental restructuring	Emotional consequences; self-assessment of affective consequences; social support (emotion); reduce negative emotions	Motivational factsheets, e.g., hunger vs craving, emotional eating, etc.; social support through lifestyle advisors & group members Enjoyable physical activity/exercise to reduce negative emotions; inspirational food diary
Reflective Motivation	Intentions	Education, Persuasion, Enablement	Action planning; barrier identification	Overcoming barriers; decisional balance; meal planning; choosing healthy meals; motivational texts; branded items
	Beliefs about capabilities	Education, Persuasion, Enablement	Verbal persuasion to boost self-efficacy; focus on success	Building confidence (body image and self-esteem); enhancing self-efficacy through physical activity
	Beliefs about consequences	Education, Modelling	Vicarious reinforcement	Online videos; group sessions
	Goals	Education, Enablement	Goal setting (outcome); goal setting (behaviour); review of outcome & behaviour goals; action planning	Progress tracking; weight loss goals; long-term goals; other appropriate & progressive goals; autonomous goals

*TDF* Theoretical Domains Framework, *COM-B* components of the COM-B model, as follows: Capability-Opportunity-Motivation – Behaviour

### Capability

#### Physical capability

Many barriers related to perceptions of physical capability were highlighted, including fatigue/exhaustion and a general lack of motivation. Reduced shoulder range of motion and safety concerns around exercise, such as exacerbating lymphoedema, were other physical capability barriers. Improved knowledge generally, and in relation to the level of control they can have over their future health and bodily changes as a result of their cancer diagnosis and treatment, was seen as an important step in overcome barriers associated with perceptions of reduced physical capability. In addition, the provision of evidence-based exercise efficacy and safety knowledge (in relation to control/attenuation of treatment-related side-effects), coupled with appropriate goal progression and peer engagement in team challenges were discussed as strategies for building physical capability confidence.

#### Psychological capability

An important psychological barrier to dietary and physical activity behaviour change after breast cancer was a previous failure to achieve sustainable weight loss. Comfort eating, exhaustion/feeling tired, and feeling low emotionally were other factors influencing dietary behaviours. However, participants had a broad range of aspirations which they hoped could be realised through participation in an appropriately delivered weight management intervention. Aside from their desire to experience successful weight loss, participants a need for more self-confidence, self-esteem and contentment, having greater reassurance about the lifestyle behaviours they were engaging in (and about the future), and gaining improved knowledge and control of their health and bodily changes experienced as a result of their cancer diagnosis and treatment.

### Opportunity

#### Physical opportunity

Preferences for dietary and exercise support, as a means of helping to enable behaviour change, were explored in the co-design workshops. It was felt that flexible dietary options would help to make dietary behaviour change more manageable. Participants wanted diet options to choose from (via example recipes and meals), cooking skills guides and suggested that food/meal taster sessions would be a useful component of the dietary component of the intervention.

A requirement for regular and frequent exercise support was also emphasised but avoiding complicated exercise modalities, such as some aerobics routines, that can make women feel self-conscious. A need for qualified motivational instructors (to instil confidence) was also highlighted and there was a feeling that on-line videos would be helpful for demonstrating correct technique (particularly upper-body exercises). The women stressed that exercise support sessions should cover the effects of adjuvant treatments such as chemotherapy on exercise capacity, as well as how to balance an exercise programme against the debilitating effects of cancer-related fatigue. It was felt that the latter should be addressed early in the intervention, together with a formal assessment of comfortable range of motion.

Traditional exercise spaces were unpopular amongst participants, as they felt intimidating. A particular dislike for mirrored walls in the gym environment was expressed, because of the heightened sense of self-consciousness this can engender. In contrast, community venues and halls, and local university facilities were suggested as the most acceptable and accessible locations for hosting the intervention. No particular time of day was preferable for everyone, and the best compromise was to run both daytime evening support sessions. For participants in employment, it was suggested that a referral letter from a General Practitioner or Oncologist could help to facilitate attendance via release from work.

#### Social opportunity

The social and group aspect of a future weight management/lifestyle intervention was discussed, and with some differences emerging regarding how this should be managed. Participants expressed a preference for attending support sessions with small to medium sized groups of women perceived as being “similar” to them, as this would provide the ideal platform for sharing common experiences and peer-to-peer support. Some participants expressed concerns about being placed in a support group with younger, fitter women, as this would make them feel uncomfortable. However, others were less concerned about this and felt that the shared experiences of breast cancer and treatment would be enough to form a rapport with other women of all ages.

The use of peer role models and previous success stories as part of the support sessions was also viewed positively and participants wanted to maintain good links with their treating hospital and general practitioner so that intervention support sessions could be referred, thereby helping to overcome attendance problems due to work commitments. The idea of collecting personal success stories from “graduates” of the programme in video, audio or written form, for use in future support sessions (or as a means of engaging future patients in the support programme) arose during discussions. It was felt that a previous “graduate” of the programme talking about their positive experiences would be highly motivational and could even convince future patients to sign up to the support programme. However, participants were split on the topic of using photographs to demonstrate weight loss and improvements in body confidence amongst previous participants as a source of motivation for others.

There were discussions about whether socialising with other group members after the group support sessions and regular social media contact with other participants would be motivational. Although some participants expressed an interest in setting-up a social media support group with others in the cohort, the overall feeling was that this should be an optional component. The potential benefits of setting up a “buddy-system” was also discussed, particularly with another member of the group who lived nearby. This could also provide an opportunity to share travel to the support sessions. Participants had mixed views on this, and in accordance with the overarching desire for a flexible programme, it was agreed that individual choice should be respected regarding the level of support needed. For example, if participants wanted to opt out of additional social support elements, it would be accepted by others without affecting group rapport.

### Motivation

#### Automatic motivation

Emotional or comfort eating, exhaustion/feeling tired, feeling low emotionally and a general lack of motivation, reduced shoulder range of motion and exercise safety concerns are likely to be important barriers to automatic motivation in this context. The inclusion of easily accessible factsheets explaining common treatment-related side-effects and providing tips on how to overcome low levels of motivation was seen as a potentially useful strategy for addressing this barrier. Useful topics were considered to be: *For days when you don’t feel like cooking—quick, simple ingredient recipes*; *For days when you've lost motivation, what to do to get back on track with eating well and exercising (including “what works for me” tips from other cancer survivors)*; *Activities to suit your mood/symptom (including an evidence-based explanation of why specific activities can help)*; *I need a drink*; *Tired and Hungry*; *Pushed for time*; *Feeling good today*; *Feeling like eating super healthy*; *Got time to spend cooking for me and others*; *Links to credible websites for further information*.

#### Reflective motivation

Participants felt that setting challenges and/or targets within the support group (and possibly using a buddy system for support) would be motivational. However, it was stressed that goals needed to be appropriate and relevant for members of the support group, such that an element of choice and flexibility was possible. Example challenges that were suggested included walking a set number of miles per month, trying out one new thing every day/week/month and an inch-loss challenge over a set period of time (e.g., waist/hip circumference measure), building-up to planking for a given duration over a set period of time, etc. Group versus group competitive challenges (with different support groups of women) and the concept of competing and comparing with other teams/cohorts was not popular amongst the participants.

Participants expressed their preference for an introductory session with the instructor at the beginning of the programme to discuss and set a personalised weight loss target. In addition, regular weigh-ins were regarded as important for maintaining motivation in some participants but the feeling was that these should be optional and not conducted publicly. There was no consensus on the optimum frequency of individualised weigh-ins, which should be based on personal preference, and with no pressure to discuss their results at group-based sessions. It was agreed that a range of other physiological measurements could also be used to track progress on a personal monitoring sheet, e.g., BMI; waist circumference; fasting blood glucose and cholesterol; blood pressure; bone mineral density; upper-limb volume/lymphoedema status; muscular and aerobic fitness. There was the feeling that achievements should be acknowledged but without being over-celebratory, and that progress should be assessed in terms of achievable milestones.

Use of a visually inspiring, colourful, and illustrated food diary (using high-quality photographs) was seen as having potential to help motivation for dietary behaviour change. Space to record daily water intake was seen as being potentially useful, as was an option to record daily mood as a means of visually linking what they ate with mood, and thereby helping to identify patterns e.g., sugar crashes. Participants were familiar with monitoring dietary behaviours (e.g., portion sizes and calories) but did not want this to be too time consuming, given that they also saw the benefits of monitoring physical activity behaviours. A simple tool that could be used to quickly record dietary and physical activity behaviours as a motivational aid was seen as the preferred option.

Most participants felt that receiving motivational texts from the instructor would be beneficial, although some expressed a preference to opt-out because such texts could become irritating or intrusive if too frequent. Examples of motivational texts that were agreed upon included: *How can you get your 5,000 steps/5-a-day/try something new today?* However, there was a general preference for such text messages to include queries about how they were doing on a particular day, with the possibility of starting a two-way conversation with the instructor if needed.

A variety of branded items, including tote bags, pens, pins, stickers, arm bands, were presented to participants to gauge their opinion on whether such items would promote allegiance to the programme as a means of improving motivation. Participants were split on the potential motivational benefits of branded items but did not want any reference to cancer in the branding. Overall, it was agreed that branded items that could serve a constructive purpose as part of engagement with the intervention (e.g., tote bags, water bottles, etc.) would be useful.

### Mode of delivery and intervention content

Views expressed in the co-design workshops strongly supported the provision of evidence-based weight management education and support via the establishment of a peer-support network with expert input and facilitation. A programme of regular skills and confidence building workshops (‘Support & Skills workshops’; Fig. 2b), either in-person or remotely via video-conferencing, bolstered by bespoke and relatable (in terms of examples used and images of other women) printed and online educational resources and options for additional instructor-led/peer to peer communications was regarded as an optimal approach to delivery of the intervention. The initial counselling session provides an opportunity for BCPs to discuss their weight management goals and explore ways in which they can overcome logistical barriers to health behaviour change with the lifestyle advisor, either one-to-one or as part of a small group.

The content and organisation of the Support & Skills workshops is presented in the Supplementary Table. Module 1 is delivered during the first 2.5 months of the intervention and covers topics that were of high priority to BCPs. This period is the most intense, with fortnightly group-based sessions being led by a qualified lifestyle advisor. Module 2 begins after three months and is delivered via monthly group-based sessions, but with interpolated monthly telephone contacts. Telephone contacts between the lifestyle advisor and patients are to provide personal support and help address any questions and concerns regarding participation in the intervention. The final six months of the programme (Module 3) has the same frequency of group-based sessions and telephone contacts but the emphasis switches to building skills for long-term maintenance of health behaviour change (e.g., relapse prevention, goal setting, etc.). Group-based workshops throughout the support programme include exercise taster sessions.

BCPs requested that any cancer branding be minimised on printed support materials, as this would serve as a reminder of past experiences, rather than future aspirations. In addition, matching 2–3 women on the basis of age within each intervention cohort could help to alleviate sensitivities regarding age and perceived fitness discrepancies within the group. There was a general feeling that peer-support sessions should be non-judgemental and fun and with an atmosphere of flexibility, so that participants could opt-in or opt-out of different activities. Dovetailing the support programme with the end of primary treatment was seen as a viable implementation pathway amongst HCPs and BCPs [31].

## Discussion

Weight gain is common amongst women undergoing early-stage breast cancer treatment and has been linked to the physical and psychological impacts of a cancer diagnosis influencing motivation for healthy lifestyle behaviours, adjunctive treatments (and associated impact on dietary patterns due to taste disturbances, altered food choices, comfort eating and the perceived convenience of, or craving for, less healthy foods) and treatment-induced menopause [6, 8, 32–36]. Furthermore, studies have reported low adherence to healthy lifestyle recommendations amongst breast cancer patients and survivors [37–40], despite evidence of poorer survival outcomes in overweight women [1, 2] and the wide-ranging health benefits resulting from weight management interventions [6, 14, 37]. Hence, there is a need for interventions that can effectively promote sustained health behaviour change and long-term weight loss maintenance in women recovering from primary breast cancer treatment [41, 42]. Accordingly, a co-design approach, guided by the BCW [27], was used to develop a weight management support programme (intervention) based on modifiable determinants of health behaviour change and relevant behaviour change strategies for this patient group [16, 43].

The co-design workshops showed that BCPs would value being part of a regular series of educational support sessions with “similar others”. This is consistent with previous research, in which empathy received from women perceived to be “in the same boat” and “same as you” during group-based lifestyle interventions was regarded as instrumental in helping them move from feeling isolated to feeling accepted, while also providing subtle peer-pressure to aid adherence [7, 44]. Establishing a structure for frequent contact and social support is consistent with best-evidence strategies for promoting changes in dietary and physical activity behaviours in the general population [15] and in people living with and beyond cancer [16]. Participants expressed a preference for positively engaging with small cohorts of women enrolled onto the programme at the same time, providing a solid basis for capitalising on the motivational potential of group cohesion. Group cohesion, which is a dynamic construct strongly influenced by perceptions of unity and personal attraction to group-based tasks and social objectives, develops from team-building activities and working towards a common goal [45]. Providing a forum for small groups of women to share ideas and experiences, guided by evidence-based knowledge, and with a common purpose of developing achievable solutions to weight management challenges, is therefore highly conducive to the development of group cohesion and motivation for sustainable health behaviour change. Other ‘in-built’ intervention strategies for developing group cohesion include setting-up buddy systems [6], engaging in achievable group challenges (not team versus team), participating in social media support groups and 2-way motivational text messaging with the lifestyle advisor. Progress monitoring, hearing examples of personal success stories from programme “graduates” and using branded programme items (e.g., tote bag, water bottle, etc.) were also seen as a means of sharing successes and fostering a spirit of allegiance to the group.

Support from a qualified and empathetic lifestyle advisor via an initial consultation (either individually or as part of a small group to set a personal weight loss goal) and ongoing dialogue via the group-based sessions was seen as an important element of the intervention, in accordance with previous qualitative research [6, 7]. This was regarded as an important for developing physical and psychological capabilities in a safe environment and evidence suggests that similar instructor-led programmes of face-to-face and remotely delivered educational support have resulted in significant weight loss over periods of 3–24 months in breast cancer survivors [10–14]. The lack of access to credible, evidence-based information on lifestyle-related issues has been highlighted previously by BCPs, citing insufficient support from health professionals [8, 9], and indicating important gaps in physical and social opportunities. Thus, having access to appropriately qualified lifestyle advisors, including input from registered dieticians and exercise specialists, who have knowledge of the physical and emotional issues faced by BCPs, was seen as an important element of the intervention. In particular, BCPs felt strongly that group-based support sessions, example recipes, myth-busting sessions and exercise taster sessions should be underpinned by the most up-to-date public health guidance on weight management and physical activity. Finally, BCPs felt that the intervention should not be stringently prescribed but should allow a flexible approach to weight management behaviours (based on core principles) and to the scheduling of group-based sessions to avoid clashes with other commitments, including work [46, 47].

The emotional consequences of diagnosis and treatment were shown to strongly influence motivation for weight management behaviours [6, 8, 32–35]. Hence, an important topic of discussion in the co-design workshops was how the support programme could help to overcome motivational challenges, such as emotional and compulsive eating patterns [44]. In this regard, the availability of “user-friendly fact sheets (using non-medical language) presenting evidence-based tips and strategies to help women meet the day-to-day motivational challenges associated with weight management behaviours was a valuable and novel idea that emerged from the workshop discussions. BCPs also liked the idea of using an inspirational food diary (to include recipe ideas) to help maintain their motivation for healthy eating and having access to a bespoke programme web-platform which could provide access to all intervention educational materials and links to other credible weight management advice. Multiple delivery mediums have previously been recommended to cater for different people with different preferences, regarding how they would like to access the intervention [46]. Progress tracking and achievable goal setting were other motivational strategies discussed in the co-design workshops, in accordance with best evidence behavioural strategies [16, 43]. However, it was clear that discussions of goal setting and progress monitoring would need to be sensitively managed. For example, while some BCPs felt that regular weigh-ins would be motivational, others felt some level of reluctance to share and/or discuss their progress in the group setting.

Finally, having access to a support programme that can be delivered virtually has implications for inclusivity. The Covid-19 pandemic has affected how people can interact with each other safely and may have increased anxieties amongst vulnerable BCPs who have received immune-suppressing anti-cancer treatments [48]. Importantly, the group-based support sessions can be delivered virtually via video or teleconferencing to assist those shielding or fearful of increased risk due to their health status, or in face-to-face in community locations that allow for social distancing guidelines to be adhered to. Having a virtual delivery option enables responsivity to changing government guidance on Covid-19 and future pandemics.

### Strengths and limitations

The co-design workshops enabled the intervention to be designed by BCPs with lived experience of the physical and psychological issues impacting motivation for health behaviour change. This inclusive approach allowed for greater understanding of how the support should be structured and delivered to overweight women undergoing treatment for ER + breast cancer. The co-design process was underpinned by a strong theoretical base and shows that the COM-B/TDF model can be systematically applied to barriers and facilitators of weight management behaviours in this patient group. The broad age-range of overweight BCPs involved in the co-design workshops (35–70 years) and varied weight management experiences allowed for a diverse representation of views. A further strength is that the intervention model is capable of being delivered remotely to small groups via screen-based technologies that have gained popularity and usage since the emergence of the Covid-19 pandemic. This increases the reach and scalability of the intervention, increasing accessibility to distant and rural communities, and means that even women considered to be clinically extremely vulnerable in the context of new Covid-19 variants can participate in support group sessions. Despite these study strengths, a number of limitations have to be acknowledged. Importantly, participants recruited for the co-design workshops were a (non-random) purposively selected sample of English speaking, white women, who were not representative of ethnic minority groups. This minimal level of diversity in participants’ cultural and educational backgrounds means that important issues of relevance to underrepresented groups may have been overlooked within the co-design process. This limitation is further compounded by the developed intervention resources, which at present are only available in the English language. In addition, all participants were recruited from large patient catchment NHS Trusts in the North of England and South Yorkshire, which may limit the generalisation of findings to women in this region.

## Conclusions

In conclusion, the two-stage co-design approach enabled the development of a theoretically-informed intervention with appropriate content, structure and delivery model to address the weight management challenges faced by BCPs. Offering a route to supported lifestyle behaviour change addresses an important unmet need for women and their treating clinicians. However, because time constraints for clinical appointments make such provision with NHS cancer care pathways difficult to deliver [49], accessible and adoptable weight management support that stands apart from, but dovetails with the NHS breast cancer care pathway could offer the best option. The clinical benefits and cost-effectiveness of this co-designed support programme versus standard care now need to be robustly evaluated via an adequately powered clinical trial.

## Supplementary Information

**Additional file 1:** **Supplementary Table**. Intervention content, showing the different modules.

## Data Availability

All data generated or analysed during this study are included in supplementary file of the article.

## Abbreviations

BCW: Behaviour change wheel
BPC: Breast cancer patient
COM-B: Capability – Opportunity – Motivation – Behaviour
ER +: Oestrogen-receptor positive
HCP: Healthcare professional
NHS: National Health Service
TDF: Theoretical Domains Framework
