# Supplementary material for: Co-designed weight management intervention for women recovering from oestrogen-receptor positive breast cancer
Source: BMC Cancer. 2022 Nov 22;22:1202. doi: 10.1186/s12885-022-10287-y (PMC9682743; doi:10.1186/s12885-022-10287-y)
Supplement: Supplementary file 1 — Additional file 1: Supplementary Table. Intervention content, showing the different modules. [file 12885_2022_10287_MOESM1_ESM.docx]

**Supplementary table**: Intervention content, showing the different modules.

| Session number | Module 1  (Sessions 1-5) | Module 2  (Sessions 6-10) | Module 3  (Sessions 11-15) |
| --- | --- | --- | --- |
| 1 | **Welcome to NEWDAY^ABC^**  Introduction: evidence-based principles of Moving Well & Eating Well; goal setting  **Taster session: Moderate & vigorous physical activity** | **Recommended Daily Amounts; eating well; taste disturbances**  Reading food labels & Recommended Daily Amounts for nutritional health  **Taster session: Exercise and hot flushes** | **Moving forward after NEWDAY^ABC^ Part 1**  Contingency planning & relapse prevention: self-management beyond NEWDAY^ABC^  **Taster session: Yoga guest session** |
| 2 | **Portion sizes & eating well for Recovery**  Identification of different food groups and recommended portion sizes  **Taster session: Strength training** | **Alcohol, drinks & hydration**  Recommended alcohol guidelines; hydration; healthy drinks & more nutritious options  **Taster session: Exercise, hydration and fatigue** | **Eating out & on the move; takeaways**  Choosing healthier meals when eating out; eating on the move and ordering takeaways  **Taster session: Zumba guest session** |
| 3 | **Building Confidence**  Improving body image & self-esteem; specific advice on lymphoedema management  **Taster session: Exercise and lymphoedema (range of motion/ flexibility)** | **Eating & moving well as a family**  Educating family & friends: importance of PA in cancer recovery; support & motivation for long-term maintenance  **Taster session: Range of motion / flexibility** | **Moving forward after NEWDAY^ABC^ Part 2**  Long-term goal setting & contingency planning: self-management beyond NEWDAY^ABC^  **Taster session: Walking netball taster session** |
| 4 | **Mood & Lifestyle**  Hunger vs craving; emotional eating & snacking: strategies to reduce emotional eating & advice on alternative nutritious snacks  **Taster session: Circuits - exercise and mood** | **Meal Planning & eating well on a budget**  Planning meals & shopping lists according to budget  **Taster session: Circuits - strength and endurance** | **Moving & eating well for healthy aging**  Importance of sleep in recovery; bedtime routines to aid restful sleep; moving & eating well for active ageing; reducing risk of comorbidities & bone health  **Taster session: Group choice of exercise** |
| 5 | **Recap & Review**  Reviewing & discussion of information covered in previous 4 sessions  **Taster session: Safe & effective home exercise 1** | **Recap & Review**  Reviewing & discussion of information covered in previous 4 sessions  **Taster session: Safe & effective home exercise 2** | **Celebration & goodbyes**  Final session - Recap and review of the entire intervention content and social session celebrating the achievements of the group  **Taster session: Group choice of exercise** |
